# International efforts for improved terror preparedness: a necessity and an obligation

**DOI:** 10.1007/s00068-023-02251-7

**Published:** 2023-05-11

**Authors:** Gerhard Achatz, Dan Bieler, Axel Franke, Benedikt Friemert, the Deployment, Disaster and Tactical Surgery Section

**Affiliations:** 1 Department for Trauma Surgery and Orthopaedics, Reconstructive and Septic Surgery, Sportstraumatology, German Armed Forces Hospital, Ulm, Oberer Eselsberg 40, 89081 Ulm, Germany; 2 Department for Trauma Surgery and Orthopaedics, Reconstructive and Hand Surgery, Burn Medicine, German Armed Forces Central Hospital, Koblenz, Rübenacher Straße 170, 56072 Koblenz, Germany; 3 Department of Orthopaedics and Trauma Surgery, Heinrich Heine University Hospital Düsseldorf, Moorenstraße 5, 40225 Düsseldorf, Germany; 4 Central Clinical Management, German Armed Forces Hospital Ulm, Oberer Eselsberg 40, 89081 Ulm, Germany

The threat posed by both armed conflicts and targeted terrorist attacks unfortunately continues. The year 2022, with the onset of Russia's war of aggression on Ukraine, has sadly made this more than clear to us.

In our Focus-On Issue 08-2020 of the European Journal of Trauma and Emergency Surgery on this complex of topics, we were able to shed light on the management of mass casualties from the perspective of intra-hospital decision makers. However, the topic of terror preparedness goes further and encompasses many other areas, which will be examined in an excerpt in this second Focus-On-issue. The developments in Ukraine in particular show us that the threat in Europe has also changed considerably and has moved much closer and more intense towards us. We perceive this in our everyday life, but as well also in our clinical work and the inherent daily business. It is therefore our duty to address the issue of preparedness actively.

In their article, Tin et al. provide a very valuable illustration of the connection and the fluid transition between armed conflicts and targeted terrorist-motivated attacks, e.g., on the civilian population, and emphasize that here, too, even transnational agreements such as the Geneva Convention or other regulations or codes of conduct and legal requirements are no longer respected [1]. They highlight in detail the term counter-terrorism medicine (CTM) to examine the consequences and effects not so much on the individual injured person, but on the health system in total. In their conclusion, the authors clearly recommend that the preparation of our health systems must continue with high priority.

Hoth et al. can only underline this aspect with their contribution and have taken up the issue of terror preparedness in a comprehensive and systematic literature review [2]. In the context of the contribution presented here, the authors can identify the training of medical staff as a very central key element in the sense of strengthening health systems, if not the most important building block. Expert conferences initiated in Germany in 2017 clearly underline this aspect in its results, which the group of authors also elaborates on very well.

A result of the first expert conference in 2017 also was the inauguration of the so-called Terror and Disaster Surgical Care course (TDSC^®^)-course by the group of authors mentioned here as an option for the education and training of in-hospital key personnel and decision makers. After several years of conducting these courses, Achatz et al. can now present the first results of a scientific evaluation in their paper and show that the so-called tabletop exercise as the core element of the TDSC^®^-course can be used to convey very valuable educational content, also in the sense of a playful approach to this complex of topics [3].

The contribution by Lennquist Montan et al., who used the MACSIM^®^ system of the Medical Response to Major Incidients & Disaster (MRMI^®^)-course, which has been established over many years, calculated the treatment capacities of hospital infrastructure in mass casualty incidents. The scientific work presented in this Focus-On-issue also describes very well how valuable such approaches can be [4]. The authors show that it is also possible to use simulation models to record corresponding preparation needs and to derive them specifically for clinical facilities.

The importance of such trainings and developments is shown well by the results of Söderin et al. from Sweden [5]. A national survey among all clinics involved in emergency care has already demonstrated a good level of preparation and an increasingly emerging awareness for this topic. Overall, however, not a single hospital out of 87 was prepared for all key areas formulated according to the WHO criteria regarding terror preparedness, so that improvements are still feasible everywhere.

Finally, the contribution by Yanez et al. can also be understood to this effect. With their paper, the authors present a project that is intended to optimize the preclinical care of patients after major incidents. Thanks to extensive funding from the European Union [6]. In the first year of its work, a valuable alliance of medical personnel from the preclinical and clinical sectors (both medical and non-medical), non-professional healthcare providers and technical partners from industry has already been able to derive valuable results from this project, which is intended to optimize education, training—supported by new and innovative technical solutions—and thus ultimately the care of our patients. Politicians now seem to be willing to support and finance the corresponding preparation!

In summary, we may once again conclude that further preparation and improvement of our health systems is becoming increasingly important. The extensive efforts of individual groups are already showing very valuable results, and political developments indicate increasing support. However, even closer cooperation between the individual initiatives would probably enable even more valuable synergy effects and should—as the preparations for this very valuable Focus-On issue have shown—be striven for in any case!

Dear colleagues, we wish you many interesting and valuable suggestions while reading this issue and would like to thank all authors very much, not only for their contributions, but also for the work behind them and the related efforts to improve our health systems to ward off terror!

## Data Availability

For this paper no further data were evaluated.
